# Paeonol Suppresses Bladder Cancer Progression via Apoptotic Pathways: Insights from In Vitro and In Vivo Studies

**DOI:** 10.3390/ph18040472

**Published:** 2025-03-27

**Authors:** Lu Ying, Ruolan Chen, Rui Guo, Youfeng Liang, Mingxuan Hao, Xiaoyang Chen, Wenjing Zhang, Changyuan Yu, Zhao Yang

**Affiliations:** 1 College of Life Science and Technology, Innovation Center of Molecular Diagnostics, Beijing University of Chemical Technology, Beijing 100029, China; yl_tlm@163.com (L.Y.); chen13120171322@126.com (R.C.); guorui60902@163.com (R.G.); liangyoufeng@163.com (Y.L.); mssjclnl@163.com (M.H.); chenxiaoyang0225@163.com (X.C.); zhangwenjing0818@163.com (W.Z.); 2 College of Life Science and Technology, State Key Laboratory Incubation Base for Conservation and Utilization of Bio-Resource in Tarim Basin, Tarim University, Alar 843300, China

**Keywords:** bladder cancer, paeonol, apoptosis, cell cycle arrest, p53

## Abstract

**Background/Objectives**: Bladder cancer (BC), a highly heterogeneous and mutation-prone malignancy, remains a significant therapeutic challenge due to its propensity for recurrence, metastasis, and drug resistance. Natural products, particularly paeonol, a bioactive compound derived from Moutan Cortex in traditional Chinese medicine, have shown promising potential in cancer therapy. This study aims to evaluate the anti-BC effects of paeonol and elucidate its underlying molecular mechanisms. **Methods**: In vitro experiments were conducted using T24 and J82 BC cell lines to assess paeonol’s effects on cell viability, migration, apoptosis, and cell cycle progression via CCK-8, scratch, flow cytometry, RT-qPCR, and Western blot analyses. In vivo efficacy was evaluated using a xenograft mouse model, with tumor growth monitored and histopathological analysis performed. **Results**: Paeonol significantly inhibited BC cell proliferation and migration in a dose- and time-dependent manner, with IC_50_ values of 225 μg/mL (T24) and 124 μg/mL (J82) at 48 h. It induced apoptosis and arrested the cell cycle at the G1 phase, accompanied by upregulation of pro-apoptotic proteins (BID, BAX, BIM, and p53). In vivo, paeonol reduced tumor volume and weight without histopathological abnormalities in vital organs. **Conclusions**: Paeonol exhibits potent anti-BC activity by apoptotic pathways and by arresting the cell cycle at the G1 phase and inhibiting tumor growth. Its favorable safety profile and multi-target mechanisms highlight its potential as a promising therapeutic candidate for BC. These findings provide a foundation for further clinical development of paeonol-based therapies.

## 1. Introduction

Bladder cancer (BC) ranks among the top ten most prevalent cancers worldwide, characterized by high heterogeneity, frequent gene mutations, and a propensity for recurrence, metastasis, and drug resistance [1,2]. According to the GLOBOCAN 2022 database, global epidemiological surveillance data reveal a substantial BC burden in 2022, with 613,791 new cases (constituting 3.1% of all new cancer diagnoses) and 220,349 mortality events (accounting for 2.3% of cancer-related deaths), with significant epidemiological heterogeneity across populations [3]. BC demonstrates profound gender disparities, with males exhibiting a 4-fold higher age-standardized incidence (1.05%) and mortality rate (0.28%) compared to females (incidence: 0.26%; mortality: 0.07%) [3]. Genome instability, a hallmark of cancer [4], renders cancer cells particularly susceptible to developing resistance when exposed to single-target therapies [4,5]. As the third most mutation-prone cancer after lung cancer and melanoma [6,7], BC presents significant challenges for targeted therapy. At the time of initial diagnosis, approximately three-quarters of bladder cancer cases are classified as non-muscle-invasive bladder cancer (NMIBC), while 20–25% progress to muscle-invasive bladder cancer (MIBC), and a small subset (5%) present with metastatic involvement. Contemporary therapeutic strategies for BC, including surgical resection, intravesical BCG immunotherapy, cisplatin-based chemotherapy, immune checkpoint inhibitors, molecular-targeted therapies, and antibody–drug conjugates, are limited in efficacy and associated with high costs, making BC the most expensive malignancy to treat [8]. These modalities are hampered by high recurrence rates, with 31–78% of NMIBC relapsing within five years [9,10], whereas MIBC variants exhibit substantially higher recurrence rates exceeding 50% [11], largely due to intrinsic and acquired resistance mechanisms necessitating innovative strategies to overcome multidrug resistance and improve cost–efficacy ratios. Notably, the 5-year survival rate (just under 80%) for BC has remained stagnant since 1985 [2], underscoring the urgent need for novel therapeutic approaches.

Natural products, with their diverse chemical structures and biological activities, have historically served as a rich source of drug discovery [12]. Amid the rising economic burdens associated with conventional cancer therapies, plant-derived bioactive compounds—recognized as a green anticancer weapon—are emerging as cost-effective adjunctive therapeutic strategies to enhance treatment accessibility and sustainability [8,13]. Recent research demonstrates that natural compounds synergize with conventional chemotherapy regimens to enhance antitumor efficacy, overcome multidrug resistance, and exert organoprotective effects against chemotherapy-induced organotoxicity [14]. The potential cost-effectiveness advantage of natural compounds may stem from their capacity to reduce chemotherapeutic dose requirements through synergistic interactions while concurrently decreasing supportive care expenditures via attenuation of treatment-related toxicities. Targeting unconventional pathways with natural compounds offers a promising strategy to overcome drug resistance and minimize adverse effects [15,16]. Traditional Chinese medicine (TCM), in particular, has garnered attention for its potential in cancer therapy. Among its many bioactive compounds, paeonol (CAS: 552-41-0, C_9_H_10_O_3_), the principal active component of Moutan Cortex, has demonstrated remarkable antioxidant [17,18], anti-inflammatory [19], anticancer [20,21,22,23,24], neuroprotective [17], and antimicrobial properties [18]. Paeonol exhibits potent antitumor activity across various cancer types, both in vitro and in vivo [20], through multifaceted mechanisms, including induction of apoptosis [25], cell cycle arrest, and inhibition of angiogenesis. Its low toxicity and unique chemical structure further highlight its potential as a candidate for targeted cancer therapy [26,27].

Despite these promising attributes, the anti-BC mechanisms of paeonol remain poorly understood [28]. This study aims to systematically evaluate the therapeutic potential of paeonol through comprehensive in vivo and in vitro experiments while elucidating its molecular mechanisms at the cellular and molecular levels.

## 2. Results

### 2.1. Paeonol Inhibits Proliferation and Migration of BC Cells

**Figure 1:**
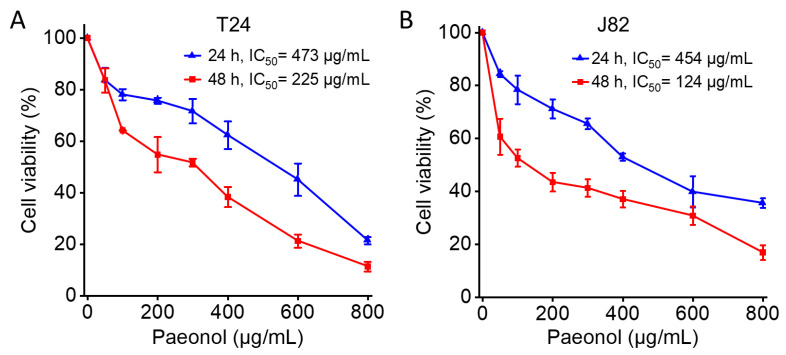
Antiproliferative effects of paeonol (50–800 μg/mL) on human bladder cancer cells assessed by the CCK-8 assay. (**A**) T24 and (**B**) J82 cell lines were treated with the indicated concentrations of paeonol for 24 and 48 h. Data represent the mean ± standard deviation (SD, *n* = 3).

To evaluate the effect of paeonol on BC cell viability, T24 and J82 cells were treated with varying concentrations of paeonol (50–800 μg/mL) for 24 and 48 h, followed by the CCK-8 assay. The IC_50_ values for T24 and J82 cells were 473 μg/mL and 454 μg/mL at 24 h, respectively (Figure 1). After 48 h of treatment, the IC_50_ values decreased to 225 μg/mL for T24 cells and 124 μg/mL for J82 cells (Figure 1). These results demonstrate that paeonol significantly suppresses BC cell viability in a dose- and time-dependent manner.

**Figure 2:**
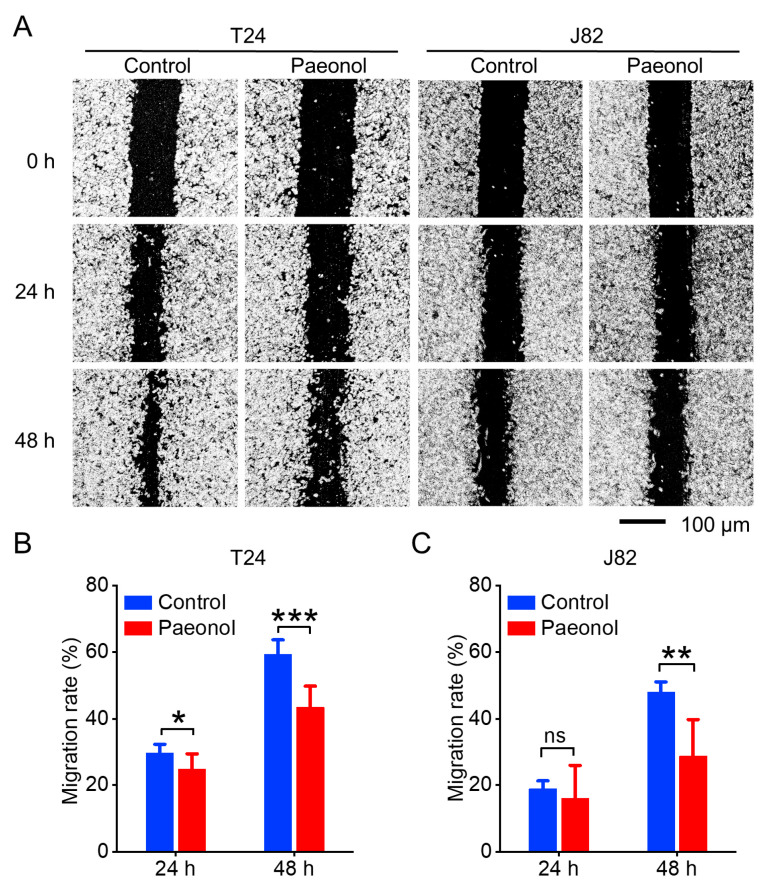
Inhibitory effects of 50 μg/mL paeonol on bladder cancer cell migration assessed by a wound healing assay for 24 h and 48 h, respectively. (**A**) Representative migration images of T24 and J82 cells. Quantitative analysis of T24 (**B**) and J82 (**C**) cell migration rates. Data are expressed as mean ± SD (*n* = 3). Statistical significance was defined as * *p* < 0.05, ** *p* < 0.01, and *** *p* < 0.001, while “ns” indicated non-significant results (*p* > 0.05). Scale bar indicates 100 μm.

To further assess the impact of paeonol on BC cell migration, a wound healing assay was performed. After 24 h of treatment with 50 μg/mL paeonol, the migration rate of T24 cells decreased from 29.66 ± 2.64% to 24.77 ± 4.66%, while J82 cells showed a reduction from 18.87 ± 2.42% to 16.03 ± 9.93% (Figure 2A–C). At 48 h of treatment with 50 μg/mL paeonol, the migration rate of T24 cells was further reduced from 59.34 ± 4.34% to 43.37 ± 6.39%, and J82 cells exhibited a significant decrease from 47.98 ± 3.05% to 28.69 ± 11.03% (Figure 2A–C). These findings indicate that paeonol effectively inhibits the migration of BC cells, with varying degrees of efficacy across different cell lines.

### 2.2. Paeonol Induces Apoptosis and Arrests Cell Cycle at the G1 Phase on BC Cells

**Figure 3:**
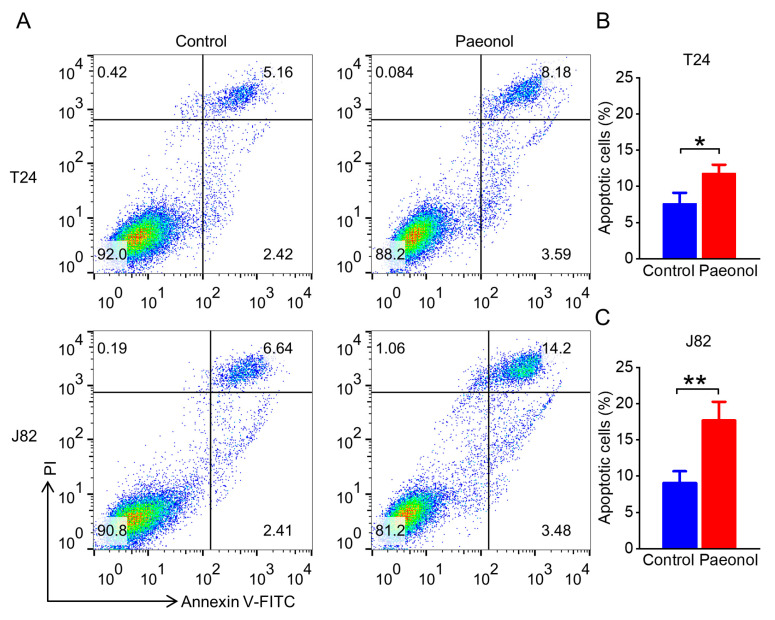
Pro-apoptotic effects of 200 μg/mL paeonol on bladder cancer cells analyzed by flow cytometry for 48 h. (**A**) Representative flow cytometry plots of T24 and J82 cells. Quantitative analysis of apoptotic rates in T24 (**B**) and J82 (**C**) cells. Data are expressed as mean ± SD (*n* = 3). Statistical significance was defined as * *p* < 0.05 and ** *p* < 0.01.

To examine the effect of paeonol on apoptosis in BC cells, T24 and J82 cells were treated with 200 μg/mL paeonol for 48 h and analyzed by flow cytometry. The apoptosis rate of T24 cells increased significantly from 7.65 ± 1.38% to 11.72 ± 1.30% (Figure 3A,B), while J82 cells exhibited a more pronounced increase from 9.03 ± 2.50% to 18.01 ± 1.95% (Figure 3A,C). These results suggest that paeonol inhibits BC cell proliferation by promoting apoptosis.

**Figure 4:**
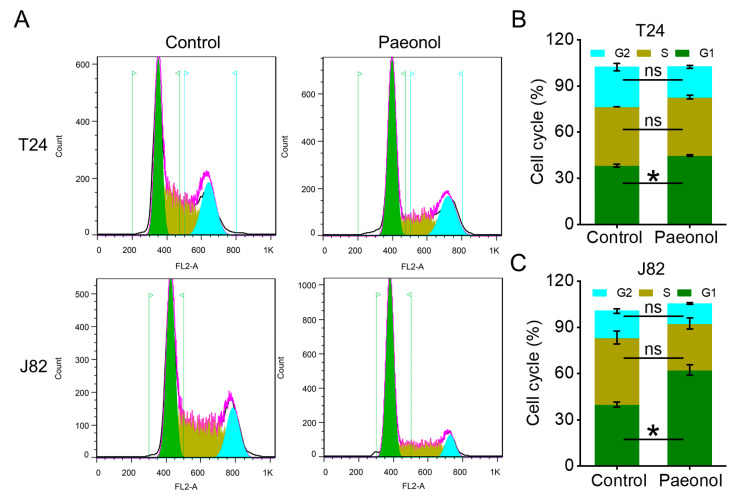
Cell cycle distribution of bladder cancer cells treated with 200 μg/mL paeonol for 48 h. (**A**) Flow cytometry histograms of T24 and J82 cells. Quantitative analysis of cell cycle phases in T24 (**B**) and J82 (**C**) cells. Data are expressed as mean ± SD (n = 3). Statistical significance was defined as * *p* < 0.05, while “ns” indicated non-significant results (*p* > 0.05).

To further explore the impact of paeonol on the cell cycle, T24 and J82 cells were treated with 200 μg/mL paeonol for 48 h and subjected to flow cytometry. In T24 cells, the proportion of cells in the G1 phase increased significantly from 38.33 ± 0.94% to 44.87 ± 0.48%, while the S phase fraction slightly decreased from 38.26 ± 0.02% to 37.88 ± 1.19%, and the G2 phase fraction declined from 25.68 ± 2.38% to 19.74 ± 1.01% (Figure 4A,B). Similarly, in J82 cells, the G1 phase population increased from 39.92 ± 1.68% to 62.17 ± 3.43%, whereas the S phase fraction decreased from 43.36 ± 4.19% to 30.24 ± 3.48%, and the G2 phase fraction reduced from 17.16 ± 1.37% to 12.92 ± 0.60% (Figure 4A,C). Collectively, these findings indicate that paeonol induces G1 phase cell cycle arrest in BC cells.

### 2.3. Molecular Mechanisms Underlying Paeonol’s Effects on BC Cells

To elucidate the molecular mechanisms of paeonol in BC cells, we investigated its effects at both the gene and protein levels.

**Figure 5:**
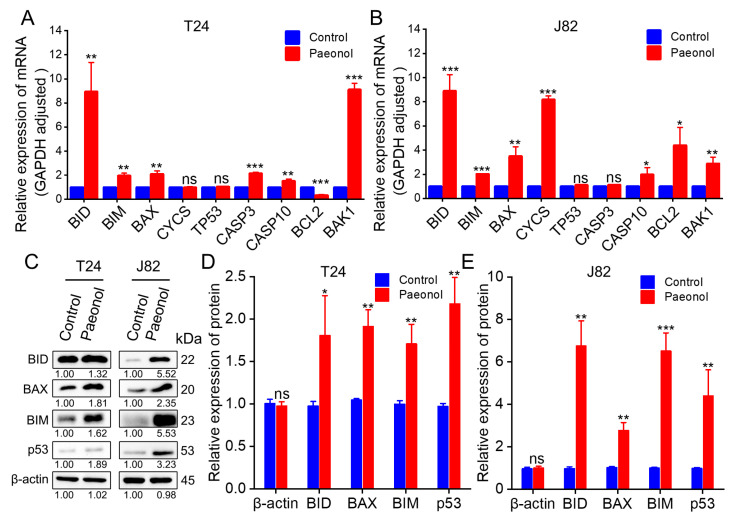
Molecular regulation of apoptosis-related genes and proteins in 200 μg/mL paeonol-treated BC cells for 48 h. Relative mRNA expression levels in (**A**) T24 and (**B**) J82 cells. (**C**) Western blot images and quantitative protein expression analysis in (**D**) T24 and (**E**) J82 cells. *GAPDH* and β-actin served as internal controls for RT-qPCR and Western blotting, respectively. Data are expressed as mean ± SD (*n* = 3). Statistical significance was defined as * *p* < 0.05, ** *p* < 0.01, and *** *p* < 0.001, while “ns” indicated non-significant results (*p* > 0.05).

At the gene level, T24 and J82 cells were treated with 200 μg/mL paeonol for 48 h, followed by RNA extraction and cDNA synthesis. RT-qPCR analysis revealed significant upregulation of *BID*, *BIM*, *BAX*, *CASP3*, *BAK1*, and *CASP10* in T24 cells, while *BCL2* expression was markedly downregulated, and no significant changes were observed in *CYCS* and *TP53* expression (Figure 5A). In J82 cells, *CASP10*, *BAX*, *BAK1*, *BID*, *BIM*, *CYCS*, and *BCL2* were upregulated, whereas *TP53* and *CASP3* expression remained unchanged (Figure 5B).

**Figure 6:**
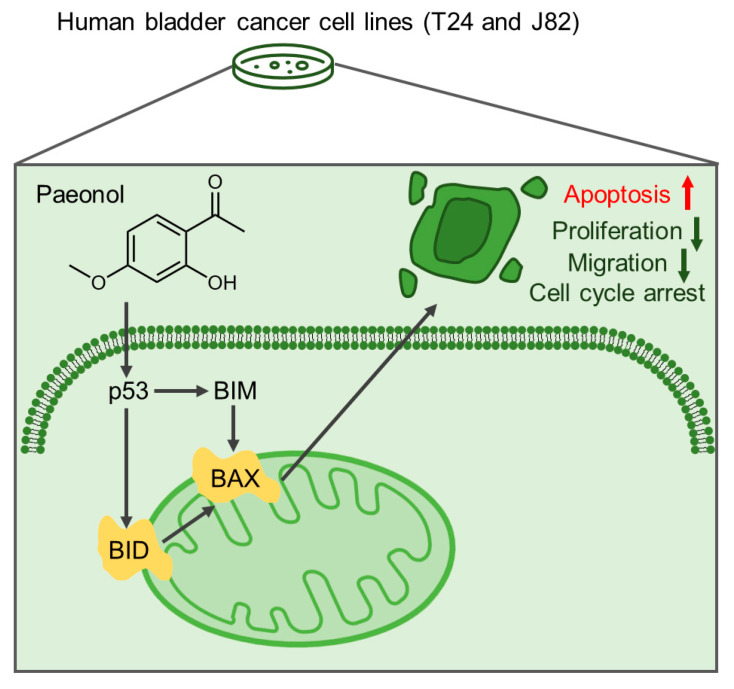
Proposed mechanism of paeonol in bladder cancer suppression. Schematic diagram illustrating p53 and pro-apoptotic proteins (BID, BAX, BIM) in the apoptotic signaling pathway.

At the protein level, T24 and J82 cells treated with 200 μg/mL paeonol for 48 h were subjected to Western blot analysis. In T24 cells, BID expression was significantly increased, while BIM, BAX, and p53 exhibited highly elevated levels (Figure 5C,D). Similarly, in J82 cells, BID, BAX, and p53 were significantly upregulated, and BIM showed a pronounced increase (Figure 5C,E). These findings indicate that paeonol upregulates key pro-apoptotic proteins (BID, BAX, BIM, and p53) in BC cells. Notably, while *TP53* mRNA levels remained unchanged, p53 protein expression was significantly elevated, suggesting that paeonol may enhance p53 activity through post-translational modifications. These results suggest that paeonol inhibits BC progression by activating the p53-mediated apoptotic pathway, leading to the upregulation of pro-apoptotic proteins, such as BID, BAX, and BIM (Figure 6). This mechanism differs from previous reports indicating paeonol’s inhibition of the PI3K/AKT pathway [28], highlighting the need for further investigation.

### 2.4. GEPIA Analysis of Gene Expression in BC Tissues

**Figure 7:**
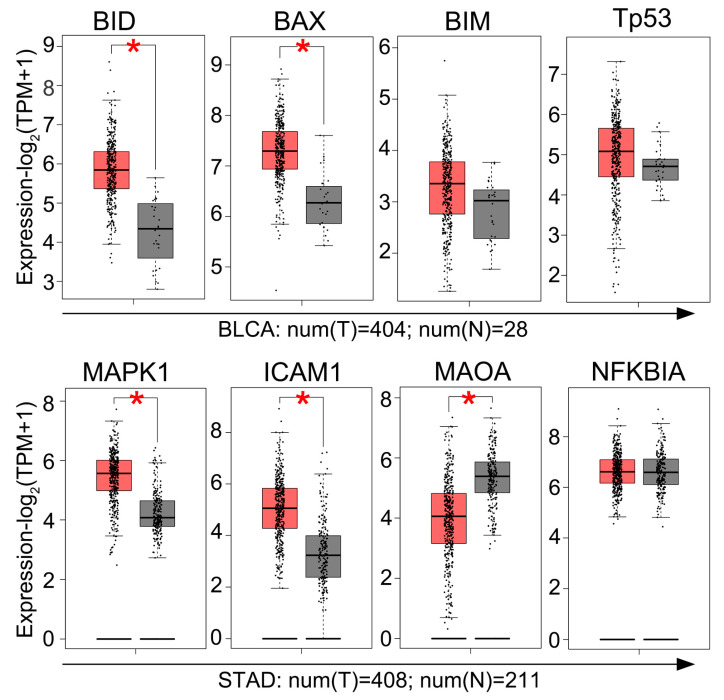
Differential gene expression analysis using the GEPIA database. Gene expression profiles in bladder urothelial carcinoma (BLCA) and stomach adenocarcinoma (STAD) tissues (red) versus normal tissues (grey). Thresholds: |Log_2_FC| > 1 with * *p* < 0.05. Database accessed via http://gepia2.cancer-pku.cn on 5 December 2024.

To further screen the potential molecular targets of paeonol in BC, we performed a GEPIA analysis (http://gepia2.cancer-pku.cn, accessed on 5 December 2024) to compare gene expression profiles between tumor tissues and adjacent normal tissues. The GEPIA analysis (Figure 7) revealed elevated *BID*/*BAX* expression in BC tissues vs. normal controls, whereas *BIM*/*TP53* showed comparable baseline levels. Importantly, the observed paeonol-induced upregulation of BIM and total p53 protein levels observed in BC cells (Figure 5C–E) represents a pharmacodynamic response distinct from intrinsic tumor biology. This distinction aligns with established models where natural compounds reactivate latent apoptotic potential independent of baseline gene expression profiles. Collectively, these results indicate that paeonol suppresses BC progression through apoptotic pathways.

### 2.5. Paeonol Inhibits Tumor Growth in a Xenograft Mouse Model

**Figure 8:**
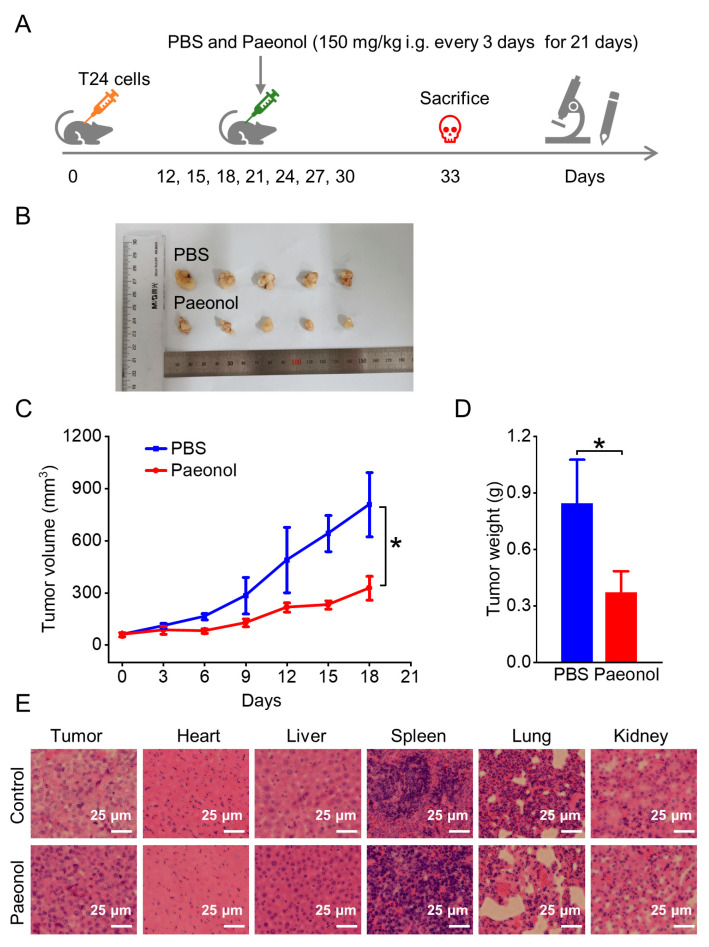
Antitumor efficacy of paeonol in xenograft models. (**A**) Experimental timeline of paeonol administration. (**B**) Representative tumor images, (**C**) tumor growth curves, and (**D**) tumor weights. (**E**) H&E staining of tumor and major organs. i.g.: intragastric administration. Data are expressed as mean ± SD (*n* = 5). Statistical significance was defined as * *p* < 0.05. Scale bar indicates 25 μm.

To assess the in vivo efficacy and safety of paeonol in BC treatment, a cell line-derived xenograft (CDX) model was established in nude mice. Tumor-bearing mice were administered paeonol (150 mg/kg) via intragastric injection (i.g.) every 3 days for 21 days (Figure 8A). After seven doses, the subcutaneous tumor volume and tumor weight in the paeonol-treated group were significantly reduced compared to the control group (Figure 8B–D), demonstrating the potent antitumor activity of paeonol in vivo. Histopathological examinations of liver, kidney, heart, spleen, and lung tissues were performed to screen for gross morphological alterations. The histopathological analysis of key organs (heart, liver, spleen, lung, and kidney) revealed no significant morphological or structural changes in paeonol-treated mice (Figure 8E). These results collectively demonstrate that paeonol exhibits significant anti-BC efficacy, with favorable safety profiles, highlighting its potential for clinical translation.

## 3. Discussion

In this study, we systematically investigated the anti-BC effects of paeonol both in vitro and in vivo. Our results demonstrated that paeonol significantly inhibited BC cell proliferation and migration while inducing apoptosis and cell cycle arrest. Furthermore, paeonol exhibited potent anti-BC activity in a human cell-derived xenograft (CDX) model, highlighting its potential as a promising therapeutic agent for BC.

Paeonol exerts inhibitory effects on multiple tumor types, including BC, glioma, apatinib-resistant gastric cancer, colorectal cancer, and pancreatic cancer, as shown in Table 1. After a 48 h paeonol treatment, the IC_50_ values of cancer cells ranged from 35 μg/mL to 401 μg/mL, and the apoptotic rates varied between 11.72% and 33.30% (Table 1). In our study, the agent exhibited differential responses, with IC_50_ values of 124 μg/mL and 251 μg/mL, corresponding to apoptotic rates of 18.01% and 11.72% (Table 1). Paeonol treatment exhibited differential effects across tumor cell lines: BC cells demonstrated moderate proliferation inhibition and apoptosis induction, gastric and pancreatic cancer cells showed high sensitivity, while glioma cells displayed minimal response. The observed variation in paeonol’s therapeutic efficacy across different tumor types may potentially arise from its differential interactions with specific molecular targets within distinct cancer cell populations [4]. The relatively modest therapeutic efficacy of paeonol observed in bladder cancer treatment may be associated with the disease’s inherent genomic heterogeneity and elevated tumor mutational burden (TMB) [6,7]. Notably, bladder cancer ranks as the third most mutation-prone human malignancy following lung cancer and melanoma, with this pronounced mutational landscape substantially contributing to the complexity of targeted therapeutic development for this neoplasm [6,7].

Notably, in our study, paeonol induced G1 phase arrest, a mechanism consistent with its anti-BC activity. This finding aligns with the work of [29], who reported a similar G1 phase arrest in the context of paeonol’s anti-glioblastoma effects (Table 1). Such targeted intervention at the G1 phase offers a compelling strategy to curb BC progression, paving the way for further exploration of paeonol’s multifaceted anticancer properties.

Apoptosis, a form of programmed cell death, plays a critical role in maintaining cellular homeostasis, and its dysregulation is a central factor in carcinogenesis [31]. Apoptosis is mediated by two primary pathways: the intrinsic pathway, which involves mitochondrial and endoplasmic reticulum signals, and the extrinsic pathway, initiated by extracellular death ligands [31]. The p53 signaling pathway is pivotal in cellular regulation, particularly in tumor suppression, cell cycle regulation, apoptosis, metabolic regulation, autophagy, and genomic stability [32]. As a master tumor suppressor, p53 transcriptionally activates a network of genes that orchestrate anticancer responses [32]. Effective anticancer therapies often induce DNA damage and eliminate cancer cells through p53-dependent mechanisms [33].

This multi-target action is consistent with the characteristic of TCM, which often involves multiple targets and signaling pathways. Indeed, relevant studies have confirmed that paeonol exerts antitumor effects through multiple targets and pathways [30], including the apoptosis signaling pathway, PI3K/AKT signaling pathway, NF-κB/p65 signaling pathway, LINC00665/miR-665/MAPK1 axis, and TGF-β1/Smad-mediated epithelial-mesenchymal transition (EMT) (Table 1). Notably, our findings suggest that paeonol may potentially activate BIM expression and modulate p53 post-translational modifications, a mechanism that has not been previously reported and contrasts with existing studies in the anticancer field (Table 1). While BCL2 is generally downregulated in antitumor responses, our study observed low BCL2 expression in the T24 cell line and high BCL2 expression in the J82 cell line (Figure 5A,B). This discrepancy may be attributed to the high heterogeneity of bladder cancer cells [2], underscoring the complexity of their molecular profiles and responses to therapeutic interventions.

Paeonol exhibits multi-target effects in the treatment of various cancers (Table 1). For instance, in gastric cancer cells treated with 60 μg/mL paeonol, the expression of MAPK1 protein was downregulated, while ICAM1 protein expression showed no significant change [30]. However, the GEPIA analysis revealed that both *MAPK1* and *ICAM1* were highly expressed in gastric cancer tissues, whereas *MAOA* expression was relatively low (Figure 7). These findings suggest that *MAPK1* may serve as a potential novel target of paeonol in gastric cancer treatment. Nevertheless, the core target of paeonol in BC remains unclear, as identifying key protein targets of monomeric compounds derived from TCM remains a major challenge in TCM-based antitumor research. Despite these challenges, successful examples such as gambogic amide and artemisinins demonstrate the feasibility of this approach [16,34,35]. It is evident that, while numerous potential targets of antitumor drugs exist, identifying the key targets is a complex task. Therefore, further studies are required to elucidate the core targets and key signaling pathways of paeonol in BC.

The potent antitumor activity and favorable biosafety profile of paeonol suggest that it has potential as a promising candidate for clinical development. However, challenges, such as poor water solubility and high effective concentrations (mg/mL), limit its clinical application [36]. Molecular modification (also known as derivative) and the development of advanced drug delivery systems are considered primary strategies to overcome these limitations [37,38]. It is well known that the efficacy of paclitaxel has been significantly improved and its side effects have been reduced through both molecular modification and drug delivery strategies. In contrast, research on drug delivery systems for paeonol began in 2007 and remains in the developmental stage [39]. Nevertheless, future research should focus on designing paeonol-loaded drug delivery systems with high efficiency, enhanced targeting capability, improved water solubility, low dosage requirements, controlled drug release, reversed tumor multidrug resistance (MDR), and reduced toxicity. Additionally, the therapeutic efficacy of paeonol in combination with existing treatment strategies, such as gemcitabine, should be evaluated. Concurrently, further in-depth studies are needed to elucidate the molecular mechanisms of paeonol and identify its optimal therapeutic targets.

While our study focused on histopathological screening, future investigations will incorporate serum biochemistry and hematological profiling under extended dosing regimens. These analyses are planned as part of a chronic toxicity study, which is essential for translational development.

## 4. Materials and Methods

### 4.1. Paeonol Preparation and Cell Culture

Paeonol (Chengdu Desite Biotechnology Co., Ltd., Chengdu, China) was dissolved in dimethyl sulfoxide (DMSO, MP Biomedicals, LLC, Illkirch-Graffenstaden, France) to prepare a stock solution, which was stored at −80 °C until use. T24 and J82 BC cells (Procell Life Science & Technology Co., Wuhan, China) were cultured in Dulbecco’s Modified Eagle Medium (DMEM, Gibco, Shanghai, China) supplemented with 10% fetal bovine serum (FBS; Gibco, Waltham, MA, USA) and 1% penicillin–streptomycin (PS; Shanghai Yuchun Biology Science and Technology Co., Ltd., Shanghai, China). Cells were maintained in a humidified incubator at 37 °C with 5% CO_2_ and routinely passaged to ensure exponential growth.

### 4.2. Cell Viability Assay and IC_50_ Determination

Cells were seeded in 96-well plates and allowed to adhere in complete medium. After attachment, the medium was replaced with DMEM containing varying concentrations of paeonol (0, 50, 100, 200, 300, 400, 600, and 800 μg/mL) and incubated for 24 or 48 h. Following treatment, the supernatant was removed, and the cells were incubated with DMEM containing 10% CCK-8 reagent (Beyotime, Shanghai, China) for 2 h. Absorbance at 450 nm was measured using a microplate reader (MULTISKAN FC, Thermo Fisher Scientific, Vantaa, Finland). The half-maximal inhibitory concentration (IC_50_) was calculated using GraphPad Prism 8.2 software (GraphPad Software, Boston, MA, USA).

### 4.3. Cell Migration by Wound Healing Assay

Cells were seeded in 6-well plates at a density of 5 × 10⁵ cells/well and cultured until reaching confluence. A sterile 200 μL pipette tip was used to create a uniform scratch across the cell monolayer. After washing with PBS to remove detached cells and debris, the medium was replaced with fresh growth medium containing 50 μg/mL paeonol. Cells were incubated at 37 °C, and images of the scratch were captured at 0, 24, and 48 h. Scratch widths were measured using ImageJ 1.54f software (National Institutes of Health, Bethesda, MD, USA), and the migration rate (%) was calculated as follows: Migration rate (%) = [(Scratch area at 0 h − Scratch area at N h)/Scratch area at 0 h] × 100%. The scratch area was measured by ImageJ 1.54f software.

### 4.4. Apoptosis Detection by Annexin V-FITC/PI Staining and Flow Cytometry

Cells were treated with 200 μg/mL paeonol in 6-well plates for 48 h. After discarding the supernatant, the cells were washed twice with ice-cold PBS and resuspended in 195 µL Annexin V-FITC binding buffer (Beyotime, Shanghai, China). Subsequently, 5 µL Annexin V-FITC (Beyotime, Shanghai, China) was added, and the cells were incubated in the dark for 5 minutes (min). The samples were kept on ice and protected from light. Immediately prior to analysis, 10 µL propidium iodide (PI; Solarbio, Beijing, China) was added, and apoptosis cells were quantified using a BD FACS Calibur flow cytometer (BD Biosciences, San Jose, CA, USA). Data were processed using FlowJo V10 software (Tree Star, Ashland, OR, USA).

### 4.5. Cell Cycle Analysis by PI Staining and Flow Cytometry

Cell synchronization was achieved through serum starvation (24 h culture in serum-free medium) prior to 48 h paeonol treatment (200 μg/mL). Cells were harvested and fixed in prechilled 70% ethanol at −20 °C overnight. Subsequent processing included (1) two ice-cold PBS washes; (2) RNA digestion with 50 µg/mL RNase A (Solarbio, Beijing, China) under light-protected conditions (30 min, 25 °C); and (3) DNA staining with 1 μg/mL PI at 37 °C for 10 min. Cells were immediately chilled on ice before analysis with a BD FACS Calibur flow cytometer (BD Biosciences, San Jose, CA, USA). FlowJo V10 software was employed for data acquisition and cell cycle phase quantification.

### 4.6. RNA Extraction and Quantitative Real-Time PCR (RT-qPCR)

Cells were treated with 200 μg/mL paeonol for 48 h. Total RNA was isolated using the Fastpure Cell/Tissue Total RNA Isolation Kit (RC112-01, Vazyme, Nanjing, China), and cDNA was synthesized using the HiScript III 1st Strand cDNA Synthesis Kit (+gDNA wiper, R312-02, Vazyme, Nanjing, China). Gene expression levels of *BID*, *BIM*, *BAX*, *CYCS*, *TP53*, *CASP3*, *CASP10*, *BCL2*, and *BAK1* were quantified by RT-qPCR using the SuperReal PreMix Plus (SYBR Green; FP205, TIANGEN, Beijing, China). Primer sequences are listed in Table 2. *GAPDH* served as the internal reference gene, and relative mRNA expression levels (QuantStudio 1, Thermo Fisher, South San Francisco, CA, USA) were calculated using the 2^−ΔΔCt^ method.

### 4.7. Protein Extraction and Western Blot Analysis

Cells were treated with 200 μg/mL paeonol for 48 h, the medium was removed, and the cells were lysed on ice using enhanced RIPA buffer (Applygen, Beijing, China) for 30 min. The lysates were centrifuged at 12,000 rpm for 10 min, and protein concentrations were quantified using a BCA Protein Assay Kit (PC0020, Solarbio, Beijing, China). Protein samples were adjusted to equal concentrations with 5× protein loading buffer (containing DTT, Solarbio, Beijing, China) and ultrapure water, denatured at 95 °C for 15 min, and stored at −80 °C until use. Proteins were separated by SDS-PAGE and transferred to PVDF membranes (Millipore, Bedford, MA, USA) using a wet transfer system. Membranes were blocked with 5% skim milk in TBST for 1 h at room temperature, followed by incubation with primary antibodies (β-actin, BID, BIM, BAX, CASP10, BAK1, and p53; Cell Signaling Technology, Danvers, MA, USA) diluted in 5% skim milk/TBST overnight at 4 °C. After three 10 min washes with TBST, membranes were incubated with HRP-conjugated secondary antibodies (goat anti-rabbit IgG or goat anti-mouse IgG; Nakasugi Golden Bridge, Beijing, China) for 1 h at room temperature. Following three additional 5 min washes with TBST, protein bands were visualized using an Enhanced Chemiluminescence (Tanon, Shanghai, China) substrate and imaged with a chemiluminescent detection system (Tanon, Shanghai, China). Band intensities were quantified using Image J 1.54f software.

### 4.8. GEPIA Analysis of Gene Expression

GEPIA (http://gepia2.cancer-pku.cn, accessed on 5 December 2024) was used to compare gene expression profiles between tumor tissues and adjacent normal tissues. |Log_2_FC| > 1 and * *p* < 0.05 were considered statistically significant.

### 4.9. In Vivo Assay

Female BALB/c nude mice (4-week-old) were obtained from Beijing Vital River Laboratory Animal Technology Co., Ltd., Beijing, China. T24 cells were subcutaneously inoculated into the left shoulder of the mice to establish a cell line-derived xenograft (CDX) model. When tumor volumes reached approximately 50–100 mm^3^, the mice were randomly allocated into control (n = 5) and experimental (n = 5) groups. Paeonol was dissolved in 0.5% sodium methylcellulose and administered to the experimental group via i.g. (150 mg/kg body weight, 100 μL) every 3 days for 21 days [40,41]. The control group received an equivalent volume of PBS containing 5% DMSO under the same regimen. Tumor growth was monitored, and tumor volume was calculated using the following formula: Volume (mm^3^) = (length × width^2^)/2. Upon reaching a tumor volume of approximately 1000 mm^3^, the mice were euthanized humanely. Tumors were excised, weighed, photographed, and fixed in 4% paraformaldehyde for subsequent histopathological analysis using hematoxylin and eosin (H&E) staining.

### 4.10. Statistical Analysis

Statistical analyses were conducted using GraphPad Prism 8.2 software and Origin 2024b software. Data were analyzed using Student’s *t*-test, and values are presented as the mean ± SD. Each experiment was independently repeated at least three times. Statistical significance thresholds were established as follows: * *p* < 0.05, ** *p* < 0.01, and *** *p* < 0.001, with “ns” denoting non-significant results (*p* > 0.05).

## 5. Conclusions

Paeonol demonstrates significant potential as a therapeutic agent for BC by effectively delaying tumor proliferation and metastasis, inducing apoptosis, and arresting the cell cycle at the G1 phase through apoptotic pathways. Our findings highlight BIM and TP53 as promising therapeutic targets for BC. Moreover, paeonol exhibits robust antitumor activity and histopathological normalities across examined tissues, further supporting its clinical potential. These results provide novel insights into the mechanisms by which paeonol disrupts the viability and proliferative capacity of BC cells. In summary, paeonol represents a highly promising candidate for the development of innovative BC therapies.

## Figures and Tables

**Table 1:** Antitumor effect and mechanism of paeonol.

Cancer Type	Potential Targets	48 h In Vitro				Animal Model (Tumor Volume and Weight Inhibition)	Molecular Mechanism
		IC_50_ (μg/mL)	Cell Apoptosis	Cell Cycle Arrest	Cell Migration		
bladder cancer (this study)	↑: BID, BIM, BAX, p53 ↑: BID, BIM, BAX, p53	225 (T24) 124 (J82)	7.65% to 11.72% (200 μg/mL) 9.03% to 18.01% (200 μg/mL)	G1 (200 μg/mL) G1 (200 μg/mL)	↓ (50 μg/mL) ↓ (50 μg/mL)	yes (150 mg/kg i.g. every 3 days for 21 days) ND	apoptotic pathway
bladder cancer [28]	↑: BAX, CASP3, AKT ↓: BCL2 ↑: BAX, CASP3, AKT ↓: BCL2	251 (T24) 265 (5637)	7.83% to 20.47% (200 μg/mL) 5.80% to 14.07% (200 μg/mL)	ND ND	ND ND	yes (100 and 400 mg/kg/day i.g. for 14 days) ND	PI3K/AKT
glioma [29]	↑: BID, BAK, BAX ↓: BCL2 ↑: BID, BAK, BAX ↓: BCL2	401 (U87MG) 88 (U251)	3.65% to 23.90% (200 μg/mL) 4.30% to 33.30% (200 μg/mL)	G1 G1	ND ND	yes (5 mg/kg i.p. every 3 days for 35 days) ND	the mitochondria-mediated intrinsic apoptotic pathway
apatinib-resistant gastric cancer [30]	↑: miR-665 ↓: LINC00665, MAPK1 ↑: miR-665 ↓: LINC00665, MAPK1	54 (BGC-823/AP) 57 (MGC-803/AP)	ND ND	ND ND	ND ND	yes (30 and 50 mg/kg/day i.p. for 28 days) ND	LINC00665/miR-665/MAPK1 axis
colorectal cancer [26]	↑: BAX, IkBa ↓: COX-2, BCL2, IKKa, NF-Kb/p65	<120 (LoVo)	1.10% to 16.10% (30 μg/mL)	ND	ND	yes (100 mg/kg/day i.g. for 11 days)	NF-kb/p65
pancreatic cancer [22]	↑: E-cadherin ↓: N-cadherin, vimentin, TGF-β1,p-Smad2/Smad2, p-Smad3/Smad3	44 (Panc-1) 35 (Capan-1)	ND ND	ND ND	↓ (17, 25 μg/mL) ↓ (17, 25 μg/mL)	ND ND	TGF-β1/Smad, epithelial-mesenchymal-transition

ND: Not Detectable, i.g.: intragastric administration, i.p.: intraperitoneal injection.

**Table 2:** Primers.

Gene	5′-3′	5′-3′
*GAPDH*	AAGGTGAAGGTCGGAGTCAA	GGAAGATGGTGATGGGATTT
*BID*	ATGGACCGTAGCATCCCTCC	GTAGGTGCGTAGGTTCTGGT
*BIM*	CTGAGTGTGACCGAGAAG	GATTACCTTGTGGCTCTGT
*TP53*	GTTCCGAGAGCTGAATGAGG	TCTGAGTCAGGCCCTTCTGT
*BAX*	CCTTTTGCTTCAGGGTTTCA	CAGTTGAAGTTGCCGTCAGA
*CYCS*	GGTGATGTTGAGAAAGGCAAG	GTTCTTATTGGCGGCTGTGT
*CASP3*	AAGCGAATCAATGGACTCT	TGTACCAGACCGAGATGT
*CASP10*	TAGGATTGGTCCCCAACAAGA	GAGAAACCCTTTGTCGGGTGG
*BCL2*	CTAAAACCCTGCCACCTCAA	CTCAGTGCTGAGTCCATCCA
*BAK1*	TCTGGCCCTACACGTCTACC	ACAAACTGGCCCAACAGAAC

## Data Availability

The data that support the findings of this study are available from the corresponding author upon reasonable request.

## Abbreviations

The following abbreviations are used in this manuscript:

BC	bladder cancer
CCK-8	cell counting kit-8
CDX	cell line-derived xenograft
d	day
DMEM	Dulbecco’s Modified Eagle Medium
DMSO	dimethyl sulfoxide
EMT	epithelial-mesenchymal transition
FBS	fetal bovine serum
h	hours
IC_50_	half maximal inhibitory concentration
i.g.	intragastric administration
MDR	multidrug resistance
MIBC	muscle-invasive bladder cancer
min	minutes
NMIBC	non-muscle-invasive bladder cancer
PI	propidium iodide
PS	penicillin–streptomycin
PVDF	polyvinylidene difluoride
RT-qPCR	real-time quantitative PCR
SD	standard deviations
SDS-PAGE	sodium dodecyl sulfo-polyacrylamide gel electrophoresis
TCM	traditional Chinese medicine
TMB	tumor mutational burden
WB	Western blotting
